# Navigating Diagnostic Pitfalls: False Positivity in GeneXpert Mycobacterium Tuberculosis/Rifampicin Assay

**DOI:** 10.7759/cureus.62889

**Published:** 2024-06-22

**Authors:** Mahavir Bagrecha, Siri Vineeth A Ganta, Shahzad Mirza

**Affiliations:** 1 Respiratory Medicine, Dr. D. Y. Patil Medical College, Hospital and Research Centre, Dr. D. Y. Patil Vidyapeeth, Pune, IND; 2 Microbiology, Dr. D. Y. Patil Medical College, Hospital and Research Centre, Dr. D. Y. Patil Vidyapeeth, Pune, IND

**Keywords:** interferon gamma release assay, bronchoalveolar lavage, bronchoscope, gene xpert mtb/rif assay, mycobacterium tuberculosis

## Abstract

Tuberculosis (TB), which is predominantly caused by Mycobacterium tuberculosis (MTB), poses severe diagnostic hurdles, especially with pulmonary tuberculosis (PTB), which spreads by aerosols. Sputum culture, the gold standard for MTB diagnosis, is time-consuming, expensive, and easily contaminated. The GeneXpert MTB/RIF (Xpert) assay, a molecular diagnostic tool, can quickly detect MTB and rifampicin (RIF) resistance. However, the ability to identify both live and non-viable MTB DNA, for example, in patients with a previous history of pulmonary tuberculosis or sampling from a contaminated bronchoscope, can result in false positives, as demonstrated in this case series. We present three cases of PTB diagnosed with Xpert, each with no conventional TB symptoms.

## Introduction

Tuberculosis (TB), an infection caused by Mycobacterium tuberculosis (MTB), has become a major issue in terms of diagnostic challenges. Pulmonary tuberculosis (PTB) spreads easily through aerosols [1]. Sputum culture is the gold standard for MTB diagnosis. However, it is expensive, contamination-liable, labor-intensive, and time-consuming [2]. Thus, early diagnosis is critical in TB management. The GeneXpert MTB/RIF (Xpert) assay is an automated nucleic acid amplification test. It can detect MTB complex and rifampicin (RIF) resistance-associated mutations within two hours. In higher prevalence settings, Xpert has a pooled sensitivity of 69.4- 84.7% and a specificity of 98.4-98.8% [3]. Xpert, a molecular approach, can identify the deoxyribonucleic acid (DNA) of MTB (both viable and non-viable), but only viable cells can develop in culture. So, Xpert-positive does not necessarily imply viable bacilli [4]. Mycobacterial DNA and Xpert positivity can persist in the absence of culturable bacilli for years after treatment [5]. Contamination of bronchoscopes with MTB can also further lead to false positivity by the Xpert assay. In this case series, we present three intriguing cases wherein individuals tested positive for PTB using GeneXpert, despite showing no discernible clinical symptoms typically associated with tuberculosis. This scenario challenges conventional understanding and warrants a deeper exploration of possible explanations.

## Case presentation

Case 1

A 65-year-old female with no known comorbidities presented with recurrent dry cough and breathlessness for over two months. High-resolution computed tomography (HRCT) of the thorax revealed left upper lobe consolidation and mild pleural effusion. After consulting with pulmonary medicine, a bronchoscopy was performed. The bronchoalveolar lavage (BAL) sample tested positive for GeneXpert MTB, with very low detection and indeterminate rifampicin resistance. The patient was started on anti-tubercular treatment (ATT) according to her weight band. Unfortunately, ATT was continued despite the persistence of symptoms. One month later, increased breathlessness and persistent coughing led to reevaluation.

**Figure 1:**
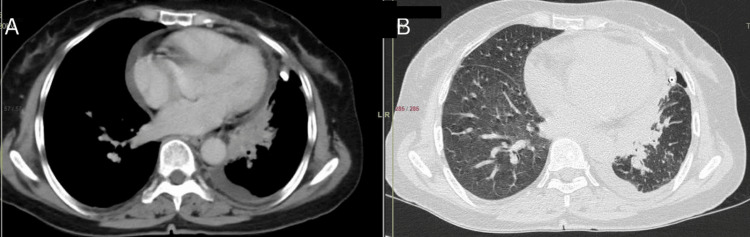
CECT thorax A: large heterogenously enhanced, ill-defined soft tissue attenuation of size 7.5 x 5 x 3 cm is noted in the left hilar and peri-hilar regions, with marked narrowing of the left upper lobe bronchus, and a left-sided pleural effusion is also seen; B: left hydropneumothorax with consolidation/mass in the hilar and peri-hilar regions CECT: contrast-enhanced computed tomography

Ultrasonography showed moderate left-sided pleural effusion, and a tube thoracostomy drained 450 ml of straw-colored fluid, which was analyzed. Contrast-enhanced computed tomography (CECT) of the thorax raised suspicion of malignancy with left-sided hydropneumothorax (Figures 1A, 1B). A second bronchoscopy revealed external compression and bronchial narrowing in the left upper lobe. An endobronchial biopsy tested positive for malignant cells, indicating adenocarcinoma, confirmed by thyroid transcription factor 1 (TTF1) positivity. The interferon gamma release assay (IGRA) was negative. Given the high negative predictive value of IGRA, ATT was discontinued, and the patient began chemotherapy for adenocarcinoma. Over the last three to four months of follow-up, the patient showed symptomatic improvement.

Case 2

**Figure 2:**
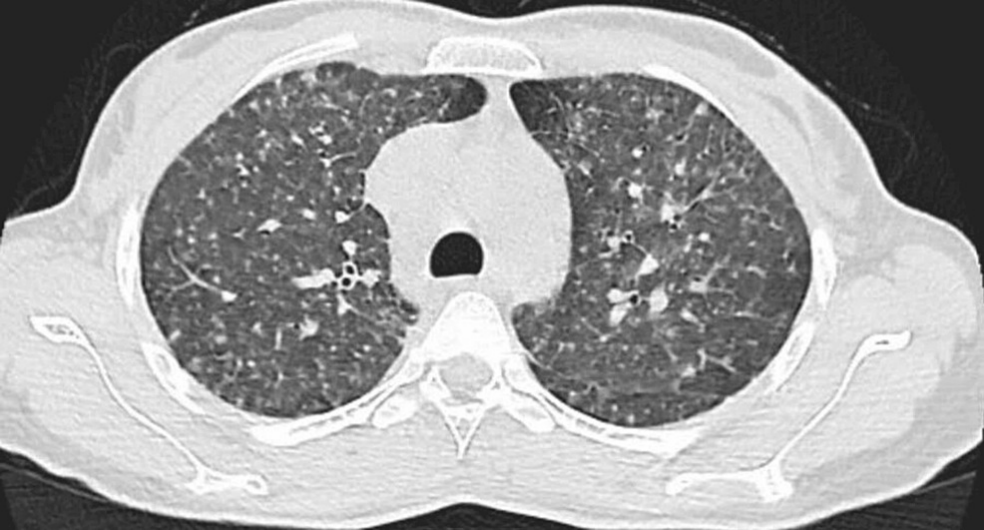
HRCT thorax Small nodular opacities scattered randomly at bilateral lung fields. HRCT: high-resolution computed tomography

A 29-year-old female, with no known co-morbidities, presented to a local hospital with complaints of cough (dry in nature), fever (intermittent and low grade), and breathlessness (MMRC grade 2) for one month. The patient was admitted and advised for a high-resolution computed tomography (HRCT) thorax, which showed small nodular opacities scattered in bilateral lungs, raising suspicion for pulmonary tuberculosis (Figure 2). Sputum for gram staining and acid-fast bacillus (AFB) was negative and hence subjected to bronchoscopy. BAL was sent for gram staining, AFB, and a Gene Xpert assay. BAL Gene Xpert was positive, showing low-detected MTB with no rifampicin resistance. The patient was started on anti-tubercular treatment. Despite being on ATT for two months, the patient's symptoms persisted, and she had repeated clinical visits with no improvement and hence visited our institution. Upon admission, the patient was disproportionately dyspneic, according to the clinical and radiological findings. A 2D echocardiography was done, which revealed mitral stenosis.

The patient was advised to undergo valve replacement surgery after a cardiologist's evaluation. The patient’s IGRA was negative. As the patient completed her intensive phase of ATT, it was decided to continue ATT while undergoing valve replacement surgery to rule out any infectious cause. The patient showed clinical and symptomatic improvement following the valve replacement surgery.

Case 3

**Figure 3:**
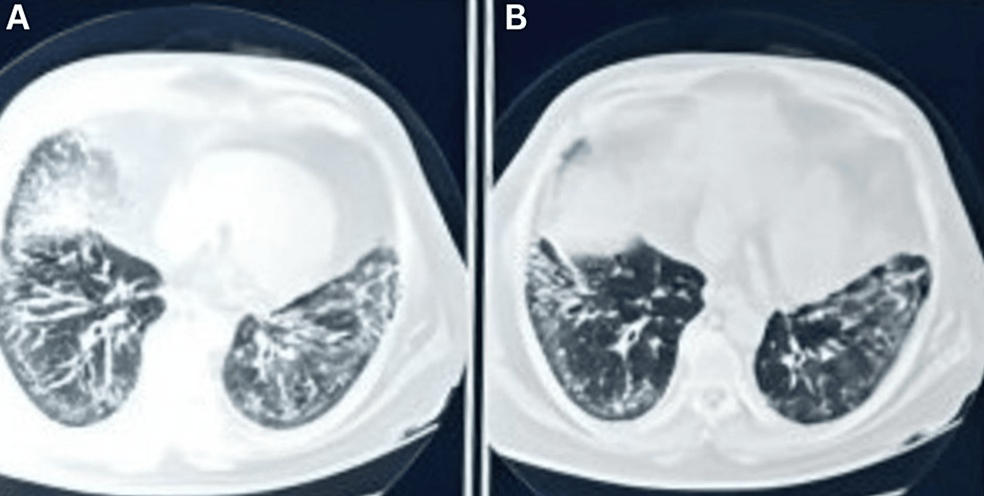
HRCT thorax A: bilateral ground glass opacities with interstitial septal thickening in bilateral lower lobes with subpleural sparing suggestive of interstitial lung disease (non-specific interstitial pneumonia pattern) and bilateral traction bronchiectasis are also seen; B: bilateral ground glass opacities in bilateral lower lobes with subpleural spacing. HRCT: high resolution computed tomograph

A 72-year-old male, hypertensive for 20 years and a chronic smoker with a smoking index of 400, presented to us with complaints of progressive dyspnea since six months (MMRC grade 3). The patient was advised for an HRCT thorax to evaluate for dyspnea, which showed interstitial septal thickening with bilateral ground glass opacities in the lower lobes suggestive of interstitial lung disease (Figures 3A, 3B). The patient underwent a bronchoscopy with a transbronchial lung biopsy (TBLB). BAL Gene Xpert came positive for MTB low detected with rifampicin resistance indeterminate. TBLB showed diffuse, moderate widening of alveolar septae with interstitial inflammation composed of lymphocytes. and moderate interstitial fibrosis suggestive of fibrotic hypersensitivity pneumonitis (HP). IGRA was negative. After taking a thorough history, the patient did not have any constitutional symptoms or radiographical evidence of TB; hence, the patient was not started on ATT.

The patient was treated with steroids and immunosuppressants for HP. The patient has been closely monitored for four months and shows no constitutional symptoms favoring TB.

## Discussion

Tuberculosis is a global public health problem, particularly in developing countries. Thus, early identification is critical for limiting mortality and transmission. Smear microscopy is a quick and inexpensive way to detect AFB, although a positive result requires at least 5,000-10,000 bacilli per milliliter of sputum. This notion makes Xpert a highly dependable alternative. Xpert can aid in the identification of smear-negative and certain culture-negative TB [5]. In recent years, IGRAs based on the T cell immune response have been demonstrated to be more sensitive and specific in the diagnosis of tuberculosis. The tuberculosis-specific T cell immunity in vitro detection approach, the γ-interferon in vitro release test (IGRA), is steadily being promoted and implemented in the clinic. This approach is unaffected by the Bacillus Calmette-Guérin (BCG) vaccine. Research by Lu Ai et al. found that IGRA has a negative predictive value of 98.7% [6]. Molecular approaches such as Xpert can detect MTB DNA (both viable and non-viable), but only viable cells grow in culture [5]. Xpert can also detect rifampicin resistance. Grant Theron et al. conducted a prospective analysis and discovered that: false-positive Xpert results occur in a significant minority of patients with previous tuberculosis; Xpert-positive, culture-negative patients remained healthy and were consistently culture-negative without treatment, indicating that they do not have active disease; and Xpert-positive results in previously treated patients were transient, with approximately half transitioning to Xpert-negative results after later retesting [7]. This study also found that patients with false-positive Xpert results had a smaller mycobacterial load than patients with true-positive Xpert results and that all patients who retested as Xpert positive but culture negative were clinically well without treatment after follow-up [7].

Other possible explanations for false positive Gene Xpert assay are relevant to bronchoscopy since it can cause both pseudo-infections and infectious breakouts [8]. The bioburden on bronchoscopes post-washing has been calculated to be roughly 6.4 x 104 colony-forming units/mL, indicating that a poorly cleaned bronchoscope is operating as a potential reservoir for contamination in both cultures and patients [9]. Shim et al. conducted an interesting investigation that shows the limitations of direct amplification testing caused by the existence of false-positive results, even when only a few dead MTB contaminate the bronchoscopes [10]. Another study by Alan H. Ramsy et al. demonstrated MTB transmission through a perforation in the bronchoscope sheath, resulting in a false-positive Gene Xpert assay [11].

In this article, bronchoscopy for all the cases was performed at different institutions. Cases 1 and 3 did not require treatment based on their radiological and clinical findings. Both cases showed no deterioration or worsening of clinical or radiological symptoms. Case 2 had already completed her intensive phase of ATT and continued with the continuation phase due to an upcoming invasive procedure. For all patients with a low-detected Gene Xpert positive result, the clinical and radiological correlation should be established before starting ATT. If bronchoalveolar lavage samples test low-positive for Gene Xpert in patients without radiological or clinical symptoms, it should raise concern about possible bronchoscope contamination.

## Conclusions

Finally, the submitted examples highlight the difficulties and potential risks of relying solely on the GeneXpert MTB/RIF assay for tuberculosis (TB) diagnosis. Despite its quickness and high specificity, GeneXpert may create false positives due to its inability to distinguish between viable and non-viable Mycobacterium tuberculosis (MTB) DNA and potential contamination, particularly from bronchoscopes. These cases demonstrate the importance of a complete diagnostic strategy that includes clinical assessments, radiological findings, and corroborative testing like the interferon-gamma release assay (IGRA). Clinicians should exercise caution when starting anti-tubercular treatment (ATT) based only on GeneXpert results without supporting clinical and radiographic data, especially when the test reveals low detection levels. Ensuring rigorous bronchoscope sterilization and taking into account the entire clinical picture can help avoid unnecessary treatments and improve patient care management. This comprehensive strategy is critical to increasing diagnosis accuracy and patient outcomes in tuberculosis management.

## References

[1] (2021). Tuberculosis. https://www.who.int//news-room/fact-sheets/detail/tuberculosis/?gad_source=1&gbraid=0AAAAACcyTy6RBLNN1ZIoCgn740f2e147a&gclid=EAIaIQobChMImfOt8oLthgMVQahmAh0YMgnuEAAYASAAEgKQgPD_BwE.

[2] Parsons LM, Somoskövi A, Gutierrez C (2011). Laboratory diagnosis of tuberculosis in resource-poor countries: challenges and opportunities. Clin Microbiol Rev.

[3] Shapiro AE, Ross JM, Yao M (2021). Xpert MTB/RIF and Xpert Ultra assays for screening for pulmonary tuberculosis and rifampicin resistance in adults, irrespective of signs or symptoms. Cochrane Database Syst Rev.

[4] Acuña-Villaorduña C, Orikiriza P, Nyehangane D (2017). Effect of previous treatment and sputum quality on diagnostic accuracy of Xpert(®) MTB/RIF. Int J Tuberc Lung Dis.

[5] Theron G, Venter R, Calligaro G (2016). Xpert MTB/RIF results in patients with previous tuberculosis: can we distinguish true from false positive results?. Clin Infect Dis.

[6] Ai L, Feng P, Chen D, Chen S, Xu H (2019). Clinical value of interferon-γ release assay in the diagnosis of active tuberculosis. Exp Ther Med.

[7] Theron G, Venter R, Smith L (2018). False-positive Xpert MTB/RIF results in retested patients with previous tuberculosis: frequency, profile, and prospective clinical outcomes. J Clin Microbiol.

[8] Culver DA, Gordon SM, Mehta AC (2003). Infection control in the bronchoscopy suite: a review of outbreaks and guidelines for prevention. Am J Respir Crit Care Med.

[9] Rutala WA, Weber DJ, and the Healthcare Infection Control Practices Advisory Committee (2019). Guideline for Disinfection and Sterilization in Healthcare Facilities.

[10] Shim TS, Chi HS, Lee SD, Koh Y, Kim WS, Kim DS, Kim WD (2002). Adequately washed bronchoscope does not induce false-positive amplification tests on bronchial aspirates in the diagnosis of pulmonary tuberculosis. Chest.

[11] Ramsey AH, Oemig TV, Davis JP, Massey JP, Török TJ (2002). An outbreak of bronchoscopy-related Mycobacterium tuberculosis infections due to lack of bronchoscope leak testing. Chest.

